# Comparison of bicuspidization and Ross procedure in the treatment of unicuspid aortic valve disease in adults – Insight from the AVIATOR registry

**DOI:** 10.3389/fcvm.2022.900426

**Published:** 2022-09-08

**Authors:** Ján Gofus, Mikita Karalko, Petr Fila, Jiří Ondrášek, Hans-Joachim Schäfers, Adrian Kolesár, Emmanuel Lansac, Ismail El-Hamamsy, Laurent de Kerchove, Christian Dinges, Jaroslav Hlubocký, Petr Němec, Martin Tuna, Jan Vojáček

**Affiliations:** ^1^Department of Cardiac Surgery, Faculty of Medicine in Hradec Kralove, University Hospital Hradec Kralove and Charles University, Hradec Kralove, Czechia; ^2^Department of Cardiac Surgery and Transplantation Brno, Faculty of Medicine, Masaryk University, Brno, Czechia; ^3^Centre of Cardiovascular Surgery and Transplantation, Brno, Czechia; ^4^Saarland University Medical Center, Homburg, Germany; ^5^East Slovakian Institute for Cardiac and Vascular Diseases, Košice, Slovakia; ^6^Institut Mutualiste Montsouris, Paris, France; ^7^Mount Sinai Hospital and Icahn School of Medicine at Mount Sinai, New York, NY, United States; ^8^Cliniques Universitaires Saint-Luc, Bruxelles, Belgium; ^9^Landeskrankenhaus Salzburg, Salzburg, Austria; ^10^Department of Cardiovascular Surgery, General University Hospital, Prague, Czechia

**Keywords:** Ross procedure, bicuspidization, reintervention, unicuspid aortic valve, aortic valve reconstruction 2

## Abstract

**Background:**

Unicuspid aortic valve (UAV) is the second most common underlying cause of aortic valve dysfunction in young adults after the bicuspid valve. The valve may be replaced (for example by pulmonary autograft) or repaired using the bicuspidization technique. The aim of our study was to compare short- and mid-term outcomes of Ross procedure with bicuspidization in patients with severe UAV dysfunction.

**Methods:**

This was a multi-center retrospective observational cohort study comparing data from two dedicated Ross centers in the Czech Republic with bicuspidization outcomes provided by AVIATOR registry. As for the Ross group, only the patients with UAV were included. Primary endpoint was mid-term freedom from reintervention. Secondary endpoints were mid-term freedom from major adverse events, endocarditis and pacemaker implantation.

**Results:**

Throughout the study period, 114 patients underwent the Ross procedure (years 2009-2020) and 126 patients underwent bicuspidization (years 2006-2019). The bicuspidization group was significantly younger and presented with a higher degree of dyspnea, a lower degree of aortic valve stenosis and more often with pure regurgitation. The primary endpoint occurred more frequently in the bicuspidization group than in the Ross group – 77.9 vs. 97.9 % at 5 years and 68.4 vs. 75.2 % at 10 years (*p* < 0.001). There was no difference in secondary endpoints.

**Conclusion:**

Ross procedure might offer a significantly lower mid-term risk of reintervention than bicuspidization in patients with UAV. Both procedures have comparable survival and risk of other short- and mid-term complications postoperatively.

## Introduction

Unicuspid aortic valve (UAV) is a congenital malformation of the aortic valve often presenting with regurgitation and/or stenosis in childhood or young adulthood (1, 2). It is the second most common underlying cause of severe aortic valve dysfunction in young adults after bicuspid aortic valve (BAV) (3, 4). The preferred treatment strategy is valve replacement with a mechanical prosthesis (mAVR) or eventually pulmonary autograft, i.e., the Ross procedure (5, 6). In some cases the valve may be repaired using the bicuspidization technique, as proposed by Schäfers et al. (7). However, this procedure is very delicate and restricted to a few centers. The long-term outcome is compromised by a substantial risk of valve failure and reintervention (8). To our knowledge, it has not been compared with other surgical strategies yet.

The Ross procedure has been systematically performed in two centers in the Czech Republic (University Hospital Hradec Kralove; Center of Cardiovascular Surgery and Transplantation Brno) since 2009, preferably in young and middle-aged adults. Patients with UAV comprise approximately one third of them. Unlike mAVR, the Ross procedure offers the patients freedom from lifelong anticoagulation and valve-related complications at the cost of long-term reintervention risk. It should be performed only in dedicated centers, similarly to bicuspidization.

AVIATOR (the Aortic Valve Insufficiency and ascending aorta Aneurysm InternATiOnal Registry) is an open valve research network administered by the Heart Valve Society (9). It comprises all essential data of patients with ascending aortic aneurysm and/or aortic regurgitation undergoing valve repair or replacement, including the bicuspidization technique. The aim of our study was to compare short- and mid-term outcomes of bicuspidization (provided by AVIATOR) with Ross procedure (data from our two centers) in adult patients with severe dysfunction of UAV.

## Materials and methods

**Figure 1:**
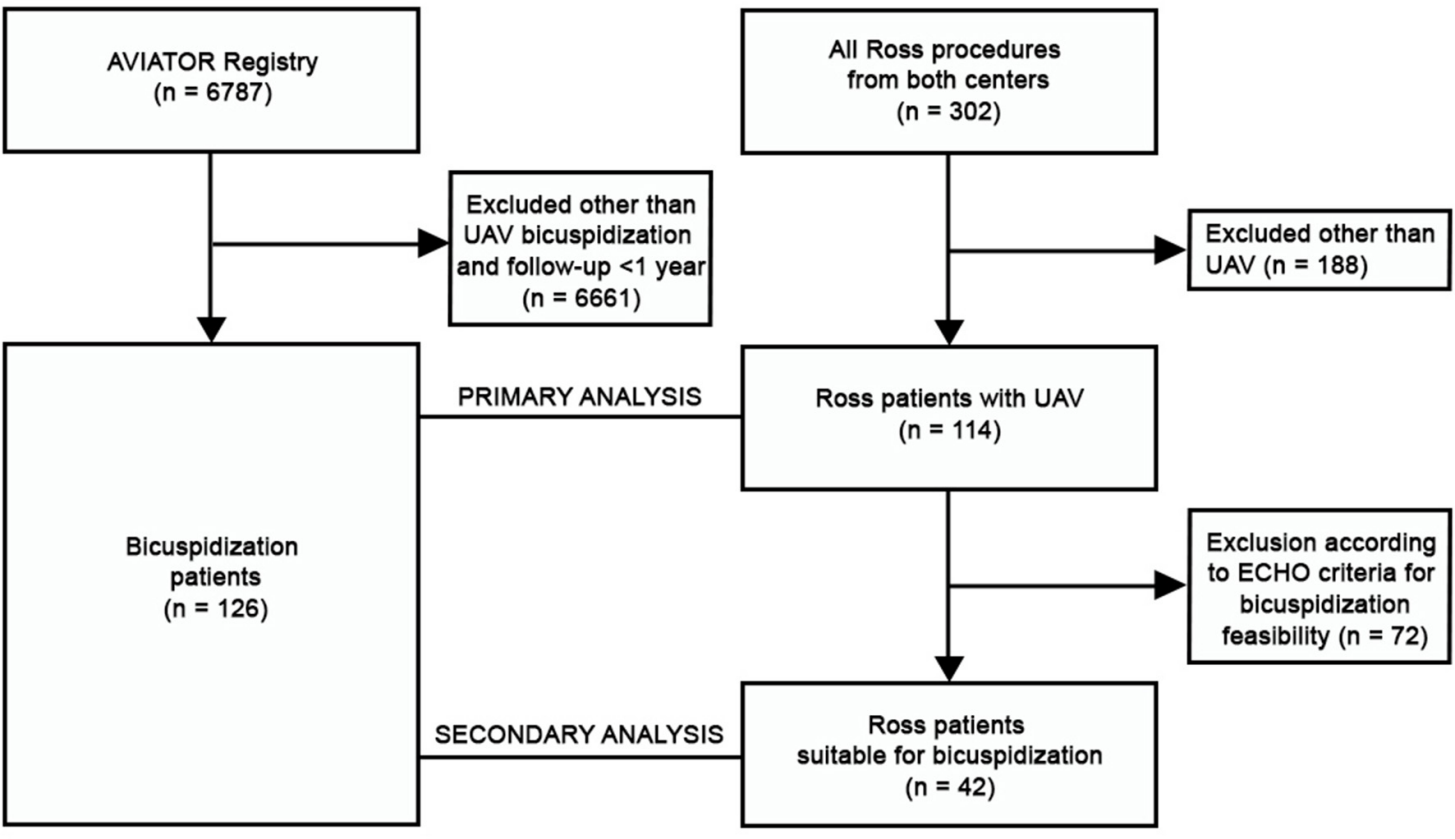
Flowchart of the study. ECHO, echocardiography; UAV, unicuspid aortic valve.

This was a multi-center retrospective observational cohort study and the flowchart is illustrated in detail in Figure 1. Data on Ross procedure were provided by the two collaborating institutions from the Czech Republic (stated above). The data were manually extracted from patient hospital records and the annual postoperative clinical and echocardiographic follow-up controls. All patients with UAV undergoing Ross procedure at our institutions were included. Although the AVIATOR registry comprises also multi-center data on Ross procedures, we decided not to utilize them for the following reasons: (i) a limited number of surgeries; (ii) restricted follow-up and limited data on reinterventions; (iii) we did not have access to preoperative echocardiograms in order to perform a thorough analysis.

The data on bicuspidization were provided by the AVIATOR registry. For this analysis, the patient inclusion criteria were as follows: (i) presence of UAV in adult patient; (ii) bicuspidization technique with any type of patch material; (iii) at least 1 year postoperative follow-up.

Patients undergoing surgery in acute settings or for infectious endocarditis were excluded from the analysis in both cohorts. The follow-up closing date was April 30, 2021. The patient′s informed consent was waived. The study was approved by the institutional ethics committees (Ethics Committee of University Hospital Hradec Králové, number 202109 P11; Ethics Committee of Center of Cardiovascular Surgery and Transplantation Brno, number 2021/84). The source data may be shared on a reasonable request addressed to the corresponding author of the study.

### Primary and secondary analysis

All Ross patients with UAV from the two collaborating institutions were included in the primary analysis. Afterwards, their preoperative echocardiograms were retrospectively reviewed by an echocardiographer (MT) and surgeons experienced in aortic valve repair (JV, PF). Only the Ross patients who could be retrospectively suitable for bicuspidization were selected for the secondary analysis. The criteria for selection were in accordance with those proposed by Schäfers et al. (10): (i) the larger part of each cusp is composed of a native cusp tissue; (ii) the corresponding cusp tissue has sufficient mobility; (iii) cusp tissue is not calcified or calcification is limited to the part resected during bicuspidization procedure. The intraoperative criteria (native cusp geometric height) could not be utilized as this selection was limited to retrospective preoperative echocardiographic review.

### Study endpoints

Primary endpoint was the freedom from reintervention in the mid-term postoperative follow-up. Both autograft and homograft reinterventions were included in the analysis of the Ross group as long as both were associated with the initial procedure. In the bicuspidization group, only aortic valve reinterventions were included in the analysis. Secondary endpoints were mid-term freedom from major adverse cardiovascular and cerebral events (MACCE; including death, stroke and hemorrhage), freedom from endocarditis and pacemaker implantation. We performed the comparison of standard short-term perioperative measures and complications, as well.

### Surgical techniques

All Ross procedures were carried out using the full root replacement technique without autograft reinforcement, as described elsewhere (11). To stabilize the proximal autograft suture line, various techniques were utilized upon the decision of the operating surgeon: inclusion of Dacron strip into the suture, extra aortic annuloplasty with a ring or PTFE stitch (12, 13). Standard cryopreserved pulmonary homograft was used in all cases (National Allograft Heart Valve Bank, University Hospital Motol, Prague).

The bicuspidization technique was performed as described elsewhere (10). Briefly, the anterior part of the leaflet adjacent to two residual commissures was resected and a new commissure was formed using various materials: autologous pericardium (fresh or preserved in glutaraldehyde), xenologous pericardium, CardioCel (Admedus, Tissue Berlin GmbH, Berlin, Germany), Matrix-auto tissue (Auto Tissue Berlin GmbH, Berlin, Germany). Ring or suture annuloplasty was added upon the surgeon's preference.

### Statistical analysis

All statistical analyses were performed with R (The R Foundation for Statistical Computing, Vienna, Austria, version 4.0.3) in RStudio (RStudio, Inc., Version 1.2.5,042). The categorical data are presented as numbers and percentages, the continuous data as mean and standard deviation. The baseline characteristics of the two cohorts were compared with the two-tailed Mann–Whitney U test for continuous variables or with the two-tailed Fisher's exact test for categorical variables. In Fisher's test for tables larger than 2 x 2 *p*-value was simulated using the Monte Carlo simulation, in 2 x 2 tables the exact *p*-value is reported. Multiple comparison correction was carried out using the Bonferroni correction.

The Kaplan-Meier survival analysis was performed using the R packages survival version 3.2-7 (Therneau, 2020) and survminer version 0.4.8 (Kassambara et al., 2020). The estimated probability of survival at pre-specified time-points is presented along with 95% confidence intervals (CI). Statistical significance of differences between survival curves was determined with a log-rank test.

## Results

Between 2009 and 2020, 114 patients with UAV underwent the Ross procedure (51 in the first institution, 63 in the second institution) and were included in the primary analysis. 42 of them were selected for the secondary analysis according to preoperative echocardiographic criteria for bicuspidization feasibility. The bicuspidization group (AVIATOR data) included 126 patients from 8 centers (see Supplementary Table 1). They were operated between 2006 and 2019. Although only adult patients were included, they were significantly younger than the Ross group (see Table 1). They presented more often with pure aortic regurgitation and lower degree of aortic valve stenosis. The diameter of the aortic root was smaller in the bicuspidization group. On the other hand, the Ross patients presented with less severe dyspnea and had higher calculated EuroSCORE II, as expected by the more complex nature of the procedure. The primary analysis showed a higher need for ascending aortic replacement in the Ross group, which did not prove in the secondary analysis. The techniques used for the stabilization of proximal autograft suture line in the Ross group as well as patch materials and annular stabilization techniques used in the bicuspidization group are provided in Table 2. The early postoperative mortality was seen only in two patients after bicuspidization (1.7%) and in no patient after the Ross procedure. The incidence of postoperative complications was generally low and comparable among the groups (see Table 3).

**Table 1:** Preoperative cohort characteristics.

	**Bicuspidization (*****n*** = **126)**			**Ross - primary analysis (*****n*** = **114)**			***p*-value**	**Ross - secondary analysis (*****n*** = **42)**			***p*-value**
Age [years]; mean, SD, *N*	28.2	9.4	93	37.9	9.1	114	**<** **0.001**	33.7	8.7	42	**<** **0.001**
BMI [kg / m2]; mean, SD, *N*	24.9	4.1	126	26.0	4.3	114	**0.019**	26.0	4.6	42	0.133
EuroSCORE II [%]; mean, SD, *N*	1.7	1.6	126	3.4	2.1	114	**<** **0.001**	2.8	1.3	42	**<** **0.001**
LVEF [%]; mean, SD, *N*	62.8	8.7	74	62.2	9.3	114	0.953	62.0	8.4	42	0.647
Aortic stenosis grade [of 3]; mean, SD, *N*	2.0	0.8	65	2.6	0.8	114	**0.000**	2.3	1.0	42	**0.015**
Aortic regurgitation grade [of 4]; mean, SD, *N*	2.8	1.0	123	2.5	1.2	114	**0.028**	3.1	0.8	42	0.102
Aortic annulus [mm]; mean, SD, *N*	26.0	4.1	73	26.6	3.0	114	0.536	27.3	3.0	42	0.113
Aortic root [mm]; mean, SD, *N*	34.0	5.9	61	37.8	5.1	114	**<** **0.001**	38.5	5.3	42	**<** **0.001**
ST junction [mm]; mean, SD, *N*	31.5	6.7	64	32.6	5.6	105	0.220	33.3	5.8	42	0.151
Ascending aorta [mm]; mean, SD, *N*	43.1	8.7	46	43.2	7.5	114	0.704	44.3	8.0	42	0.812
Female sex; *n*, %	35	27.8 %		31	27.2 %		1.00	14	33.3 %		0.557
NYHA class; *n*, %							**<** **0.001**				**<** **0.001**
*I*	29	23.0 %		50	43.9 %			20	47.6 %		
*II*	33	26.2 %		53	46.5 %			20	47.6 %		
*III*	41	32.5 %		11	9.6 %			2	4.8 %		
*IV*	5	4.0 %		0	0.0 %			0	0.0 %		
Previous cardiac surgery; *n*, %	9	7.1 %		3	2.6 %		0.142	0	0.0 %		0.114
Haemodynamic pathology; *n*, %							**<** **0.001**				**<** **0.001**
*Combined*	20	15.9 %		46	40.4 %			24	57.1 %		
*Regurgitation*	76	60.3 %		28	24.6 %			14	33.3 %		
*Stenosis*	27	21.4 %		40	35.1 %			4	9.5 %		

BMI, body mass index; LVEF, Left ventricular ejection fraction; N, number of patients with parameter at disposal; NYHA, New York Heart Association dyspnea classification; SD, standard deviation; ST, sinotubular. The bold values are statistically significant.

**Table 2:** Surgical techniques used.

	**Number of patients** **(percentage)**	**Number of reinterventions** **(percentage)**
Bicuspidization patch material	*n* = 126	*n* = 38
Autologous pericardium (glutaraldehyde)	93 (73.8 %)	30 (78.9 %)
Xeno pericardium (glutaraldehyde)	15 (11.9 %)	3 (7.9 %)
CardioCel	13 (10.3 %)	4 (10.5 %)
Autologous pericardium (fresh)	2 (1.6 %)	1 (2.6 %)
Unknown	2 (1.6 %)	0 (0 %)
Matrix-auto tissue	1 (0.8 %)	0 (0 %)
Annular stabilization in bicuspidization group	*n* = 126	*n* = 38
Ring annuloplasty (Coroneo/Dacron)	29 (23.0 %)	17 (44.7 %)
Suture annuloplasty	32 (25.4 %)	7 (18.4 %)
No stabilization	65 (51.6 %)	14 (36.8 %)
Ross annular stabilization technique – primary	*n* = 114	*n* = 6
Strip of Dacron	87 (76.3 %)	5 (83.3 %)
Extraaortic annuloplasty	15 (13.2 %)	1 (16.7 %)
No stabilization	12 (10.5 %)	0 (0 %)
Ross annular stabilization technique – secondary	*n* = 42	*n* = 3
Strip of Dacron	31 (73.8 %)	2 (66.7 %)
Extraaortic annuloplasty	6 (14.3 %)	1 (33.3 %)
No stabilization	5 (11.9 %)	0 (0 %)

**Table 3:** Perioperative and postoperative outcomes.

	**bicuspidization (*****n*** = **126)**			**Ross - primary analysis (*****n*** = **114)**			***p*-value**	**Ross - secondary analysis (*****n*** = **42)**			***p*-value**
Clamp time [minutes]; mean, SD, N	87.0	41.3	126	161.1	25.8	113	**<** **0.001**	150.9	15.7	41	**<** **0.001**
CPB time [minutes]; mean, SD, N	96.8	45.7	90	184.3	34.6	113	**<** **0.001**	188.3	23.7	41	**<** **0.001**
Ascending aortic replacement; *n*, %	58	46.0 %		75	65.8 %		**0.003**	26	61.9 %		0.108
Concomitant surgery; *n*, %	42	33.3 %		11	9.6 %		**<** **0.001**	1	2.4 %		**<** **0.001**
Type of concomitant surgery; *n*, %							**<** **0.001**				0.207
*Ascending aortic bandage*	1	0.8 %		0	0.0 %			0	0.0 %		
*ascending aortic dilation*	1	0.8 %		0	0.0 %			0	0.0 %		
*CABG*	2	1.6 %		0	0.0 %			0	0.0 %		
*Hemiarch*	1	0.8 %		2	1.8 %			1	2.4 %		
*Other*	25	19.8 %		0	0.0 %			0	0.0 %		
*Yacoub*	12	9.5 %		0	0.0 %			0	0.0 %		
Early mortality; *n*, %	2	1.6 %		0	0.0 %		0.499	0	0.0 %		1.00
Re-exploration due to bleeding; *n*, %	6	4.8 %		4	3.5 %		0.752	2	4.8 %		1.00
Transient ischemic attack; *n*, %	1	0.8 %		3	2.6 %		0.348	3	7.1 %		**0.049**
Myocardial *i*nfarction; *n*, %	0	0.0 %		0	0.0 %		NA	0	0.0 %		NA
Pacemaker implantation; *n*, %	0	0.0 %		0	0.0 %		NA	0	0.0 %		NA

CABG, coronary artery bypass grafting; CPB, cardiopulmonary bypass; MACCE, major adverse cardiac and cerebrovascular events; N, number of patients with parameter at disposal; PFO, persistent foramen ovale; SD, standard deviation; VSD, ventricular septal defect. The bold values are statistically significant.

In the primary analysis of the Ross group 6 patients underwent reintevention postoperatively. Median time to reintervention was 5.05 years. One patient with the pulmonary homograft endocarditis underwent its replacement. Second patient had a pseudoaneurysm at the distal anastomotic site of the supracoronary aortic vascular prosthesis and had it replaced. The remaining patients had a significant autograft cusp prolapse or restriction that was unacceptable for valve repair and underwent aortic valve replacement (3 mechanical and 1 biological prosthesis). Secondary analysis would have excluded 3 aortic valve replacements from the mentioned list. In the bicuspidization group 38 patients underwent reintervention over the median of 1.57 years postoperatively. One procedure was carried out in a transcatheter fashion, the rest of them were surgical. We did not have access to more detailed data regarding the nature of reinterventions in this group.

**Figure 2:**
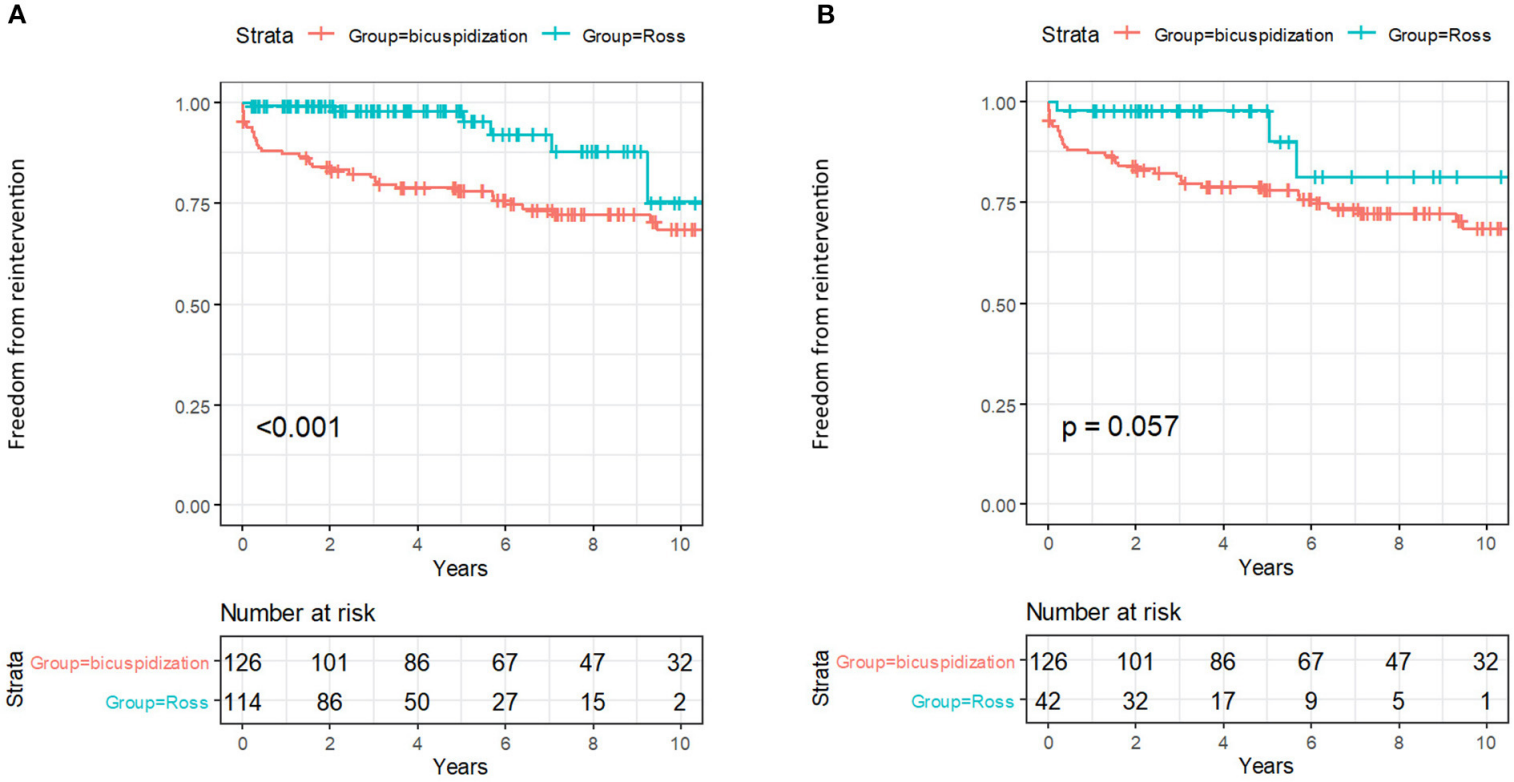
**(A,B)** Freedom from intervention in primary and secondary analysis.

Kaplan-Meier analysis showed that the mid-term risk of reintervention was significantly higher in the bicuspidization group. This was particularly true for the primary analysis, but the same trend was also seen in the secondary analysis, although marginally significant (see Figure 2). Estimated freedom from reintervention was 77.8 % (CI 70.8–80.6 %) in the bicuspidization group vs. 97.9 % (CI 95.1–100 %) in the primary analysis or 97.6 % (CI 93.1–100 %) in the secondary analysis of the Ross group at 5 years. At 10 years time-point, the freedom from reintervention was 68.4 % (CI 59.6–78.5 %) vs. 75.2 % (CI 54.1–100 %) in primary analysis or 81.1 % (CI 62.3–100 %) in secondary analysis. The type of patch material and annuloplasty did not seem to influence the risk of reintervention (Table 2). The mean clinical follow-up was longer in the bicuspidization group (8.3 years) than in the Ross group (100% complete; primary analysis 4.2 years, secondary analysis 4.1 years). The mid-term freedom from MACCE, infectious endocarditis or pacemaker implantation was extremely low in both groups and there was no significant difference among them (see Supplementary Figures 1–6 and Supplementary Tables 2–7).

## Discussion

UAV is a rare congenital aortic valve malformation present in 0.02 % of the adult population (14). In a specific subgroup of patients undergoing aortic valve surgery this may rise to approximately 5 % (3). However, even this number may be underestimated due to extensive discrepancy between echocardiographic, surgical and pathological evaluation (3, 4, 15). UAV is defined as an aortic valve with <2 fully developed commissures, while the rudimentary commissures have an abnormally low height (16). Based on the intraoperative evaluation and in accordance with this definition we observed a significantly higher amount of UAV than presented before. This was probably influenced by the fact that we analyzed a specific cohort of young and middle-aged adults indicated for aortic valve surgery. It underlines that the treatment of UAV disease comprises an important part of aortic valve surgery. A careful preoperative evaluation is crucial for choosing the correct surgical strategy.

UAVs are further divided into acommissural and unicommissural UAVs (17). The acommissural UAV is characterized by three underdeveloped, congenitally fused commissures and a “pin-hole” shaped orifice. It is usually symptomatic at birth or during childhood and is extremely rare, as reported by Roberts et al. (17). This was also observed in our Ross cohort where we saw only a single patient with this configuration. Repair of acommissural valve is extremely demanding while both commissures must be newly constructed. Only a single operation of this type was reported by the group of Schäfers (18). The bicuspidization technique was primarily developed for the second type of UAV, the unicommissural UAV. It is characterized by two underdeveloped (residual) and one normal commissure (usually located between the non-coronary and left-coronary cusp), resulting in a slit-shaped or “fish-mouth” like orifice (7). The construction of BAV from UAV is based on a rationale that BAV is usually functional until at least fourth decade of life and, moreover, aortic valve repair principles can be easily applied in this setting (8).

Unlike other treatment options (mAVR, Ross procedure), bicuspidization is performed at very few centers worldwide and its outcomes are limited to retrospective single-center studies (4, 8, 19). Out of the data provided to us by the AVIATOR, only three centers performed > 10 procedures, a single institution having performed 58 % of all cases. Comparisons with other treatment options of severe UAV disease are scarce in the literature. We found only one limited retrospective single-center comparison of bicuspidization with Ross procedure in a form of conference abstract (20). Hereby we present the first multicenter comparison of bicuspidization with other treatment, the Ross procedure in our case. Both procedures can be performed with excellent short-term outcomes. Even though the freedom from reintervention after bicuspidization was higher in our analysis than presented by Schäfers (8), it remained significantly lower than that of the Ross procedure. This was particularly true for the primary analysis of all UAVs operated, but the same trend was also preserved in the secondary analysis (*p* = 0.057). Although the median follow-up in our Ross cohort was limited, the risk of reintervention was consistent with that of other groups. Aboud et al. recently published the long-term outcomes of the Ross procedure from the multi-center European Ross Registry including almost 2 500 patients (21). They reported an average risk of reintervention of 1.3 % per patient-year over the median follow-up of 9.2 years (freedom from reintervention 95.4 % at 5 years and 84.7 % at 15 years) exhibiting similar trend to that of our cohort. Equal outcomes were observed in American population by El-Hamamsy et al. with an average risk of reintervention of 1.2 % (22).

The incidence of MACCE was extremely low and comparable in both cohorts. According to the latest evidence, the Ross procedure is the only valve substitute to offer the patients survival comparable with age- and sex-matched general population (23–25). Despite very favorable long-term outcomes in the first two decades, the most recent insight from the German Ross Registry indicates a trend toward impairment of autograft and homograft function in the third decade accompanied by a decline of survival of Ross patients (26). Although this yet remains to be proven more robustly, this evidence may provide a rationale for a postponement of valve replacement. In a selected cohort of young adults with UAV, a staged approach utilizing bicuspidization followed by later valve replacement with pulmonary autograft might be beneficial, as proposed initially in the pediatric population by Schäfers et al. (27).

### Limitations

The main limitations of our study are retrospective character and utilization of an open international registry as a data source. The length of follow-up in the Ross group was limited. Only a few patients reached the 10-year follow-up and the median follow-up was shorter than median time to reintervention which could compromise the objectivity of mid-term outcomes. However, the results were consistent with those of other groups with significantly longer follow-up and larger cohorts as mentioned above. Most of the centers performing bicuspidization had low reported procedural volume and could lead to bias of mid-term outcomes.

Despite our maximum effort to generate two equal cohorts in terms of preoperative conditions, some differences still remained in the secondary analysis. The selected Ross cohort was significantly older, had a higher degree of valve stenosis and root dilation, and reported less symptoms. These differences could lead to unwanted bias of the mid-term results.

Apart from the primary and secondary analysis, we attempted to also perform a mathematical adjustment of the retrospectively analyzed data. We chose the multivariate Cox regression analysis for the mid-term outcomes. However, the model collapsed due to high data missingness in the AVIATOR group. When adjusted for all potential confounders (age, sex, diameters of aortic annulus, root, sinotubular junction and ascending aorta, left ventricular ejection fraction), only 10 patients from the registry had all the mentioned variables at hand and could be included in calculation. This we deemed unacceptable for any objective comparison.

Double ring annuloplasty, as described by the group of Lansac et al. (28), was performed in only two patients from the bicuspidization group. This did not provide enough substrate to evaluate its influence on mid-term outcomes statistically.

We were unable to better elaborate on the comparison of mid-term postoperative valve function. The echocardiographic data provided from AVIATOR were limited, did not correlate with clinical follow-up and the incidence of reinterventions. Therefore, we decided not to present the analysis of the mid-term risk of valve failure at all. Similarly, the registry provided very limited information on the reasons for reinterventions and we could not analyze this more extensively.

## Conclusion

According to this multicenter retrospective analysis Ross procedure might offer a lower mid-term risk of reintervention than bicuspidization in patients with UAV. Both procedures exhibit a comparable short- and mid-term survival and the risk of postoperative adverse events. Bicuspidization may be used as the first step of a staged approach in the treatment of young patients with UAV, followed by the Ross procedure later on. Future studies with larger numbers of patients and eventually prospective design are necessary to provide more objective recommendations for clinical practice.

## Data availability statement

The data underlying this article will be shared on reasonable request to the corresponding author.

## Ethics statement

The studies involving human participants were reviewed and approved by Ethics Committee of University Hospital Hradec Králové, number 202109 P11; Ethics Committee of Center of Cardiovascular Surgery and Transplantation Brno, number 2021/84. Written informed consent for participation was not required for this study in accordance with the national legislation and the institutional requirements. Written informed consent was not obtained from the individual(s) for the publication of any potentially identifiable images or data included in this article.

## Author contributions

JG and MK: substantial contribution to the concept, data collection, data analysis, and drafting of the manuscript. JO and PN: critical revision of the manuscript and final approval of the version to be published. PF, H-JS, AK, EL, IE-H, LK, CD, and JH: critical revision of the manuscript, final approval of the version to be published, and data collection. MT: data collection. JV: substantial contribution to the concept, drafting of the article, critical revision of the manuscript, and final approval of the version to be published. All authors contributed to the article and approved the submitted version.

## Funding

The work was supported by the Charles University Research program Cooperatio – Cardiovascular Science.

## Conflict of interest

The authors declare that the research was conducted in the absence of any commercial or financial relationships that could be construed as a potential conflict of interest.

## Publisher's note

All claims expressed in this article are solely those of the authors and do not necessarily represent those of their affiliated organizations, or those of the publisher, the editors and the reviewers. Any product that may be evaluated in this article, or claim that may be made by its manufacturer, is not guaranteed or endorsed by the publisher.

## Abbreviations

AVIATOR: the Aortic Valve Insufficiency and ascending aorta Aneurysm International Registry
BAV: bicuspid aortic valve
CI: 95% confidence intervals
MACCE: major adverse cardiac and cerebrovascular events
mAVR: mechanical aortic valve replacement
UAV: unicuspid aortic valve.
