# Supplementary material for: Comparison of bicuspidization and Ross procedure in the treatment of unicuspid aortic valve disease in adults – Insight from the AVIATOR registry
Source: Front Cardiovasc Med. 2022 Sep 8;9:900426. doi: 10.3389/fcvm.2022.900426 (PMC9493194; doi:10.3389/fcvm.2022.900426)

**Comparison of Bicuspidization and the Ross procedure in Treatment of Unicuspid Aortic Valve Disease in Adults – Insight from the AVIATOR registry.**

**Online Supplementary Material**

**Supplementary Table 1 - Distribution of bicuspidization among centers.**

| **Bicuspidization center** | **Number of patients** | **Percentage** |
| --- | --- | --- |
| HF | 73 | 58.0 % |
| JM | 14 | 11.1 % |
| GM | 13 | 10.3 % |
| BX | 10 | 7.9 % |
| FV | 9 | 7.1 % |
| DF | 3 | 2.4 % |
| GX | 3 | 2.4 % |
| EW | 1 | 0.8 % |

**Supplementary Table 2 - Freedom from major adverse cardiac and cerebrovascular events analysis.**

| **Operation** | **time (years)** | **Number at risk** | **Number of events** | **Survival** | **Std.err** | **Lower 95 % CI** | **Upper 95 % CI** |
| --- | --- | --- | --- | --- | --- | --- | --- |
| Bicuspidalization | 2 | 118 | 5 | 0.96 | 0.0174 | 0.927 | 0.995 |
|  | 5 | 95 | 1 | 0.952 | 0.0192 | 0.915 | 0.99 |
|  | 10 | 45 | 0 | 0.952 | 0.0192 | 0.915 | 0.99 |
| Ross - Primary Analysis | 2 | 84 | 1 | 0.991 | 0.00881 | 0.974 | 1 |
|  | 5 | 37 | 0 | 0.991 | 0.00881 | 0.974 | 1 |
|  | 10 | 2 | 1 | 0.867 | 0.11615 | 0.667 | 1 |
| Ross - Secondary Analysis | 2 | 31 | 1 | 0.976 | 0.0235 | 0.931 | 1 |
|  | 5 | 13 | 0 | 0.976 | 0.0235 | 0.931 | 1 |
|  | 10 | 1 | 0 | 0.976 | 0.0235 | 0.931 | 1 |

**Supplementary Table 3 - Freedom from death analysis.**

| **Operation** | **time (years)** | **Number at risk** | **Number of events** | **Survival** | **Std.err** | **Lower 95 % CI** | **Upper 95 % CI** |
| --- | --- | --- | --- | --- | --- | --- | --- |
| Bicuspidalization | 2 | 120 | 3 | 0.976 | 0.0136 | 0.95 | 1 |
|  | 5 | 96 | 1 | 0.968 | 0.0159 | 0.937 | 0.999 |
|  | 10 | 45 | 0 | 0.968 | 0.0159 | 0.937 | 0.999 |
| Ross - Primary Analysis | 2 | 85 | 0 | 1 | 0 | 1 | 1 |
|  | 5 | 37 | 0 | 1 | 0 | 1 | 1 |
|  | 10 | 2 | 1 | 0.875 | 0.117 | 0.673 | 1 |
| Ross - Secondary Analysis | 2 | 32 | 0 | 1 | 0 | 1 | 1 |
|  | 5 | 13 | 0 | 1 | 0 | 1 | 1 |
|  | 10 | 1 | 0 | 1 | 0 | 1 | 1 |

**Supplementary Table 4 - Freedom from bleeding analysis.**

| **Operation** | **time (years)** | **Number at risk** | **Number of events** | **Survival** | **Std.err** | **Lower 95 % CI** | **Upper 95 % CI** |
| --- | --- | --- | --- | --- | --- | --- | --- |
| Bicuspidalization | 2 | 118 | 2 | 0.984 | 0.0114 | 0.962 | 1 |
|  | 5 | 95 | 0 | 0.984 | 0.0114 | 0.962 | 1 |
|  | 10 | 45 | 0 | 0.984 | 0.0114 | 0.962 | 1 |
| Ross - Primary Analysis | 2 | 85 | 0 | 1 | 0 | 1 | 1 |
|  | 5 | 37 | 0 | 1 | 0 | 1 | 1 |
|  | 10 | 2 | 0 | 1 | 0 | 1 | 1 |
| Ross - Secondary Analysis | 2 | 32 | 0 | 1 | 0 | 1 | 1 |
|  | 5 | 13 | 0 | 1 | 0 | 1 | 1 |
|  | 10 | 1 | 0 | 1 | 0 | 1 | 1 |

**Supplementary Table 5 - Freedom from thrombembolism analysis.**

| Operation | time (years) | Number at risk | Number of events | Survival | Std.err | Lower 95 % CI | Upper 95 % CI |
| --- | --- | --- | --- | --- | --- | --- | --- |
| Bicuspidalization | 2 | 120 | 0 | 1 | 0 | 1 | 1 |
|  | 5 | 96 | 0 | 1 | 0 | 1 | 1 |
|  | 10 | 45 | 0 | 1 | 0 | 1 | 1 |
| Ross - Primary Analysis | 2 | 84 | 1 | 0.991 | 0.00881 | 0.974 | 1 |
|  | 5 | 37 | 0 | 0.991 | 0.00881 | 0.974 | 1 |
|  | 10 | 2 | 0 | 0.991 | 0.00881 | 0.974 | 1 |
| Ross - Secondary Analysis | 2 | 31 | 1 | 0.976 | 0.0235 | 0.931 | 1 |
|  | 5 | 13 | 0 | 0.976 | 0.0235 | 0.931 | 1 |
|  | 10 | 1 | 0 | 0.976 | 0.0235 | 0.931 | 1 |

**Supplementary Table 6 - Freedom from infectious endocarditis analysis.**

| **Operation** | **time (years)** | **Number at risk** | **Number of events** | **Survival** | **Std.err** | **Lower 95 % CI** | **Upper 95 % CI** |
| --- | --- | --- | --- | --- | --- | --- | --- |
| Bicuspidalization | 2 | 120 | 0 | 1 | 0 | 1 | 1 |
|  | 5 | 96 | 0 | 1 | 0 | 1 | 1 |
|  | 10 | 45 | 0 | 1 | 0 | 1 | 1 |
| Ross - Primary Analysis | 2 | 85 | 0 | 1 | 0 | 1 | 1 |
|  | 5 | 37 | 0 | 1 | 0 | 1 | 1 |
|  | 10 | 2 | 1 | 0.97 | 0.03 | 0.91 | 1 |
| Ross - Secondary Analysis | 2 | 32 | 0 | 1 | 0 | 1 | 1 |
|  | 5 | 13 | 0 | 1 | 0 | 1 | 1 |
|  | 10 | 1 | 1 | 0.91 | 0.09 | 0.76 | 1 |

**Supplementary Table 7 - Freedom from pacemaker implantation analysis.**

| **Operation** | **time (years)** | **Number at risk** | **Number of events** | **Survival** | **Std.err** | **Lower 95 % CI** | **Upper 95 % CI** |
| --- | --- | --- | --- | --- | --- | --- | --- |
| Bicuspidalization | 2 | 120 | 0 | 1 | 0 | 1 | 1 |
|  | 5 | 96 | 0 | 1 | 0 | 1 | 1 |
|  | 10 | 45 | 0 | 1 | 0 | 1 | 1 |
| Ross - Primary Analysis | 2 | 85 | 0 | 1 | 0 | 1 | 1 |
|  | 5 | 37 | 0 | 1 | 0 | 1 | 1 |
|  | 10 | 2 | 0 | 1 | 0 | 1 | 1 |
| Ross - Secondary Analysis | 2 | 32 | 0 | 1 | 0 | 1 | 1 |
|  | 5 | 13 | 0 | 1 | 0 | 1 | 1 |
|  | 10 | 1 | 0 | 1 | 0 | 1 | 1 |

**Supplementary Figure 1 - Freedom from major adverse cardiac and cerebrovascular events analysis.**


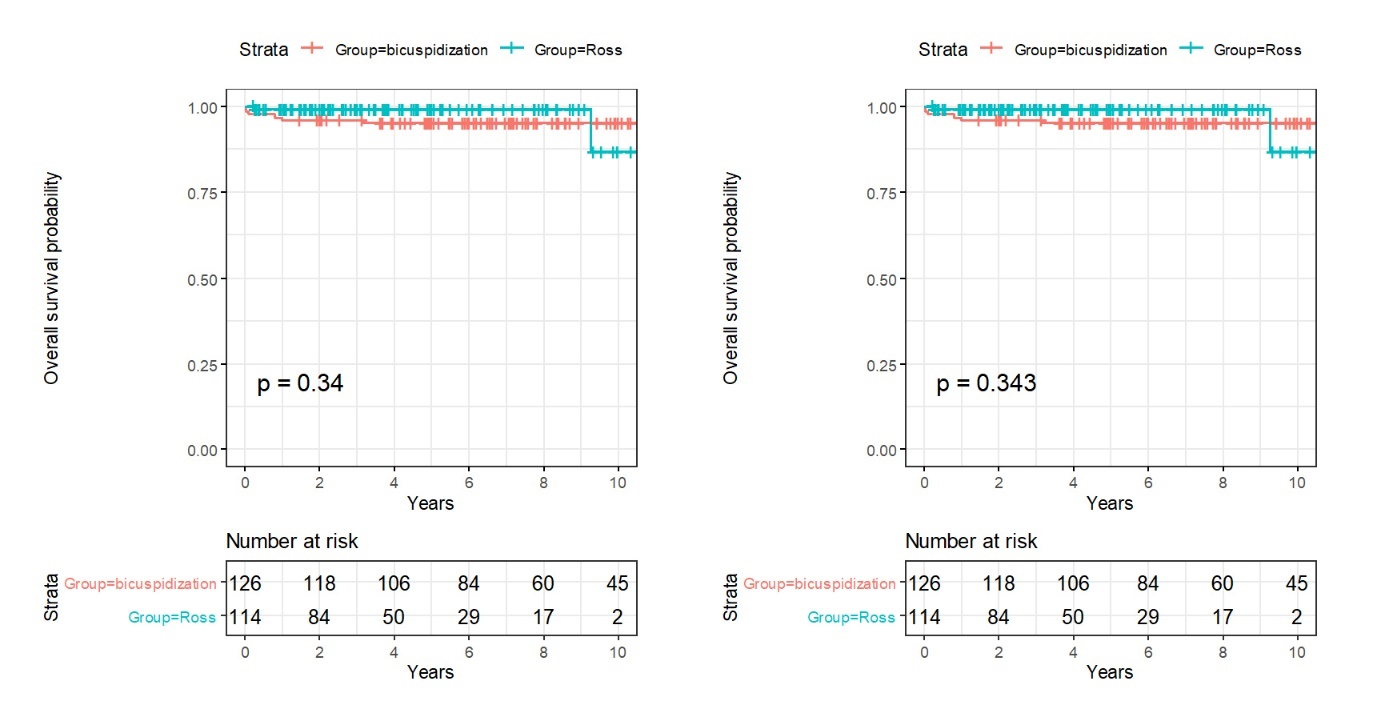


**Supplementary Figure 2 - Freedom from death analysis.**


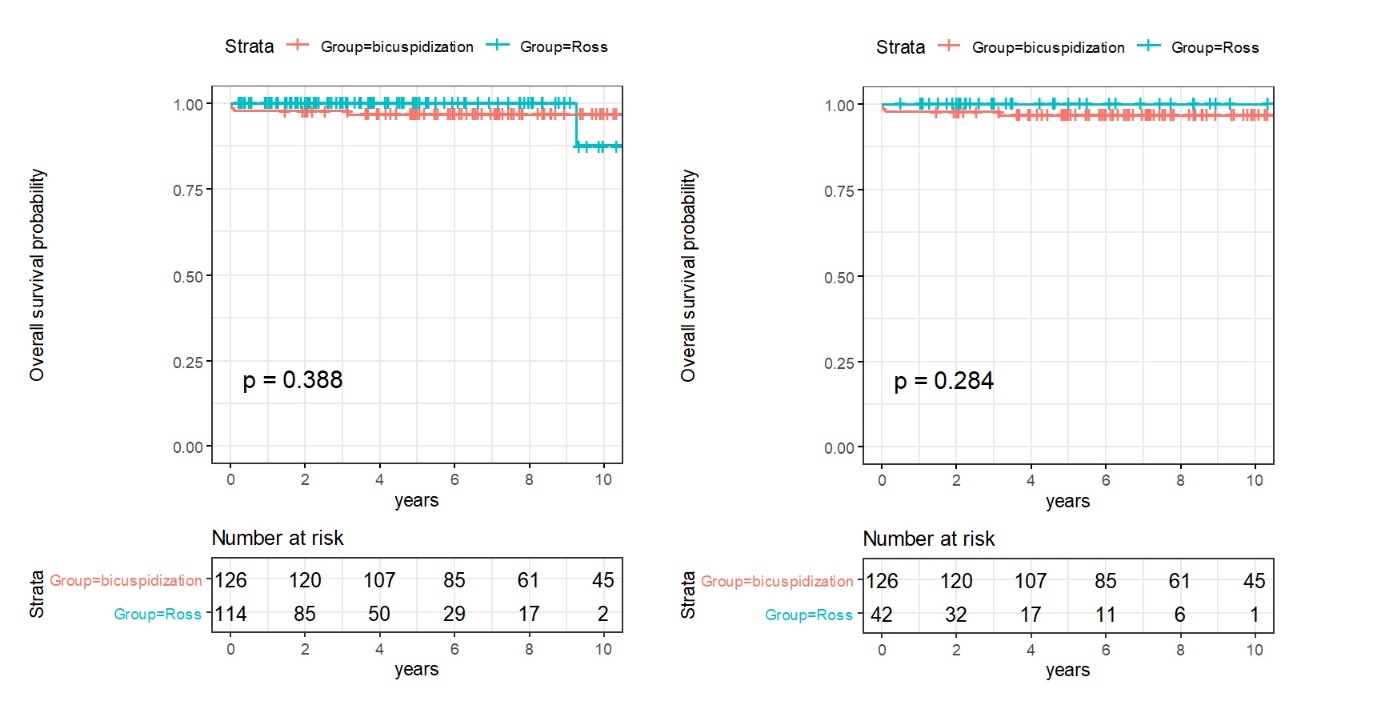


**Supplementary Figure 3 - Freedom from bleeding analysis.**


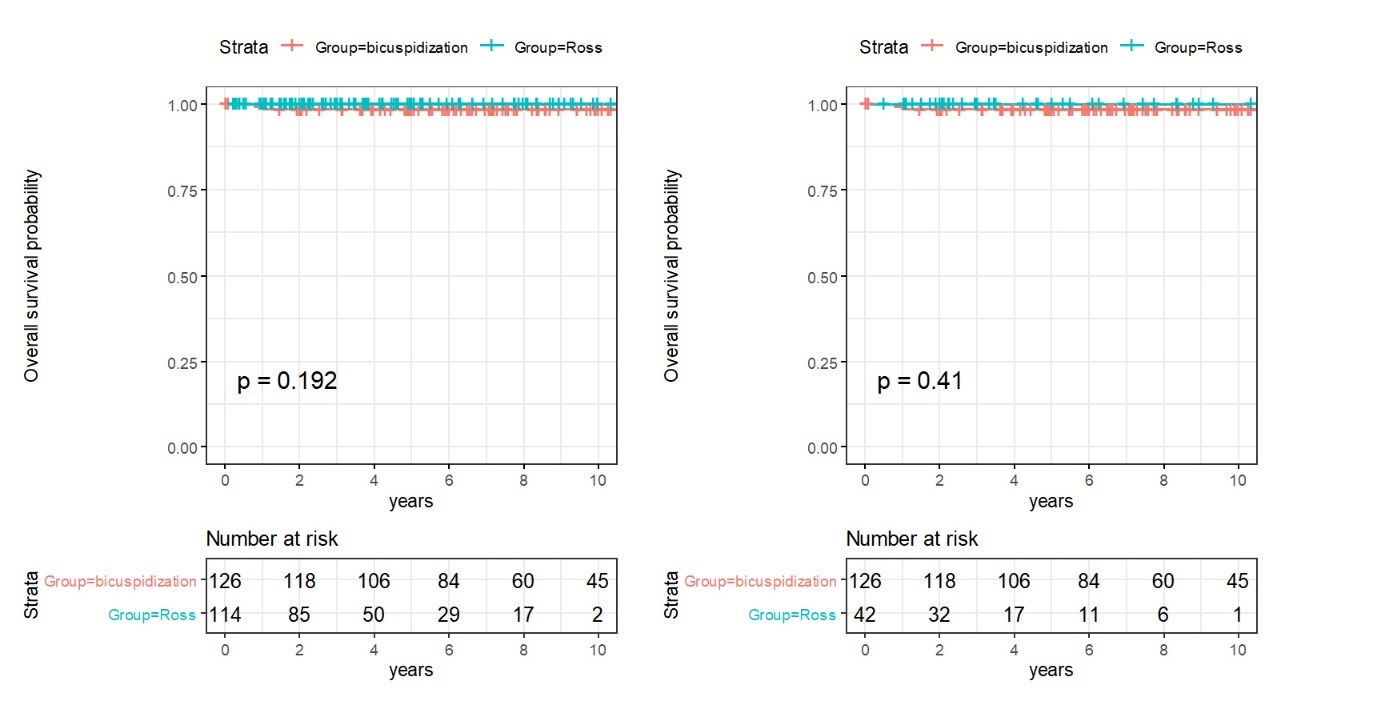


**Supplementary Figure 4 - Freedom from thrombembolism analysis.**


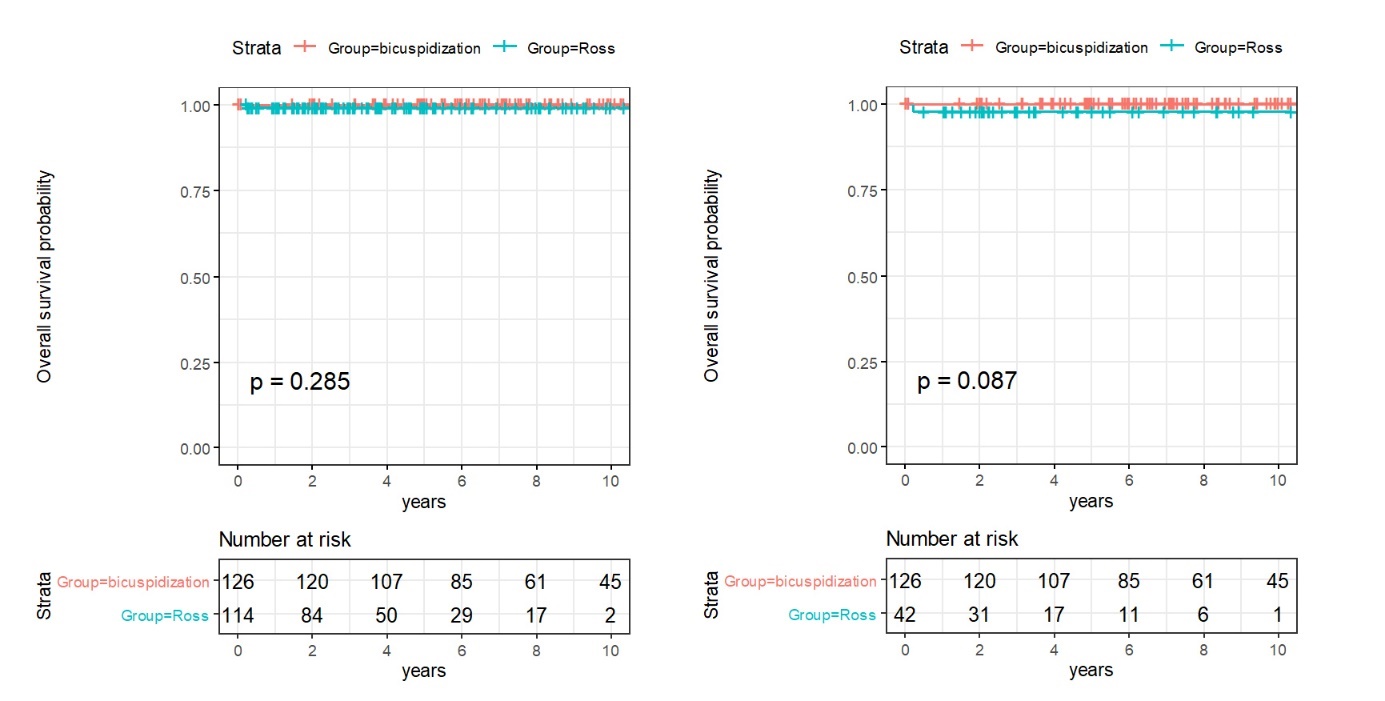


**Supplementary Figure 5 - Freedom from infectious endocarditis analysis.**


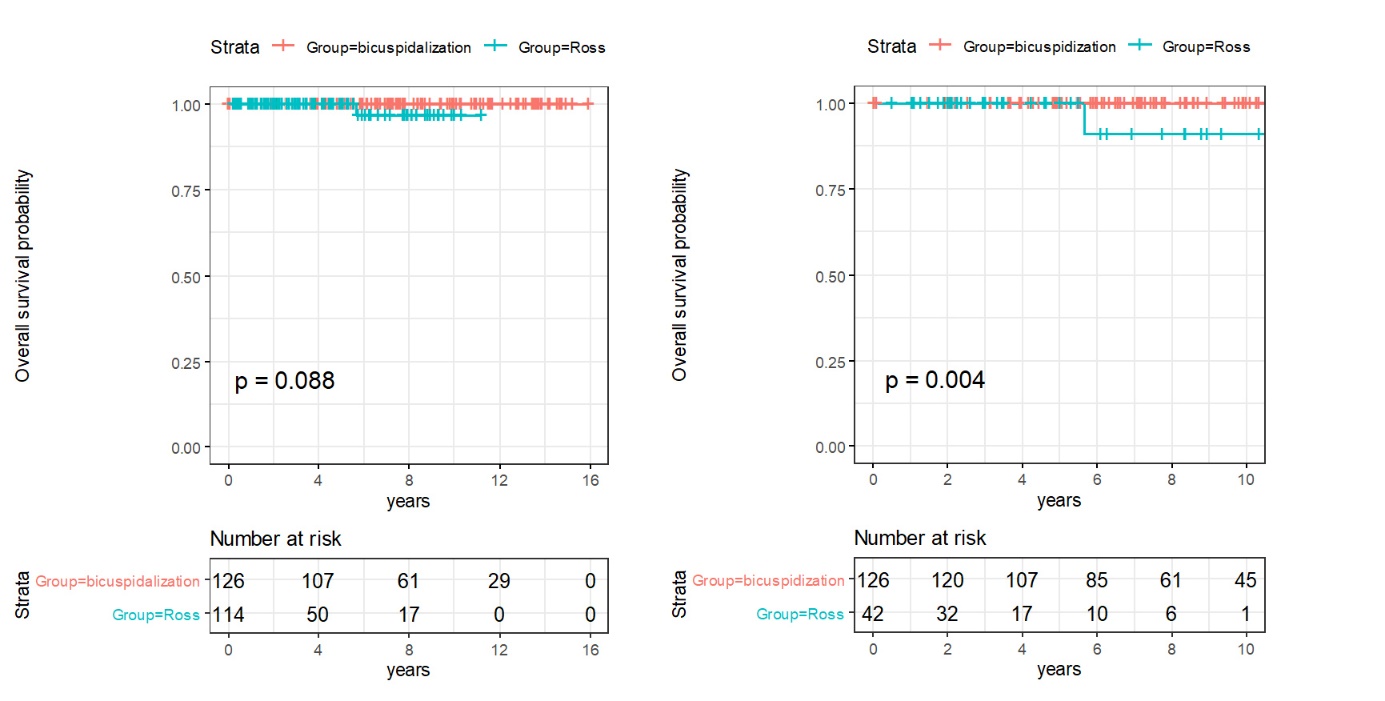


**Supplementary Figure 6 - Freedom from pacemaker implantation analysis.**


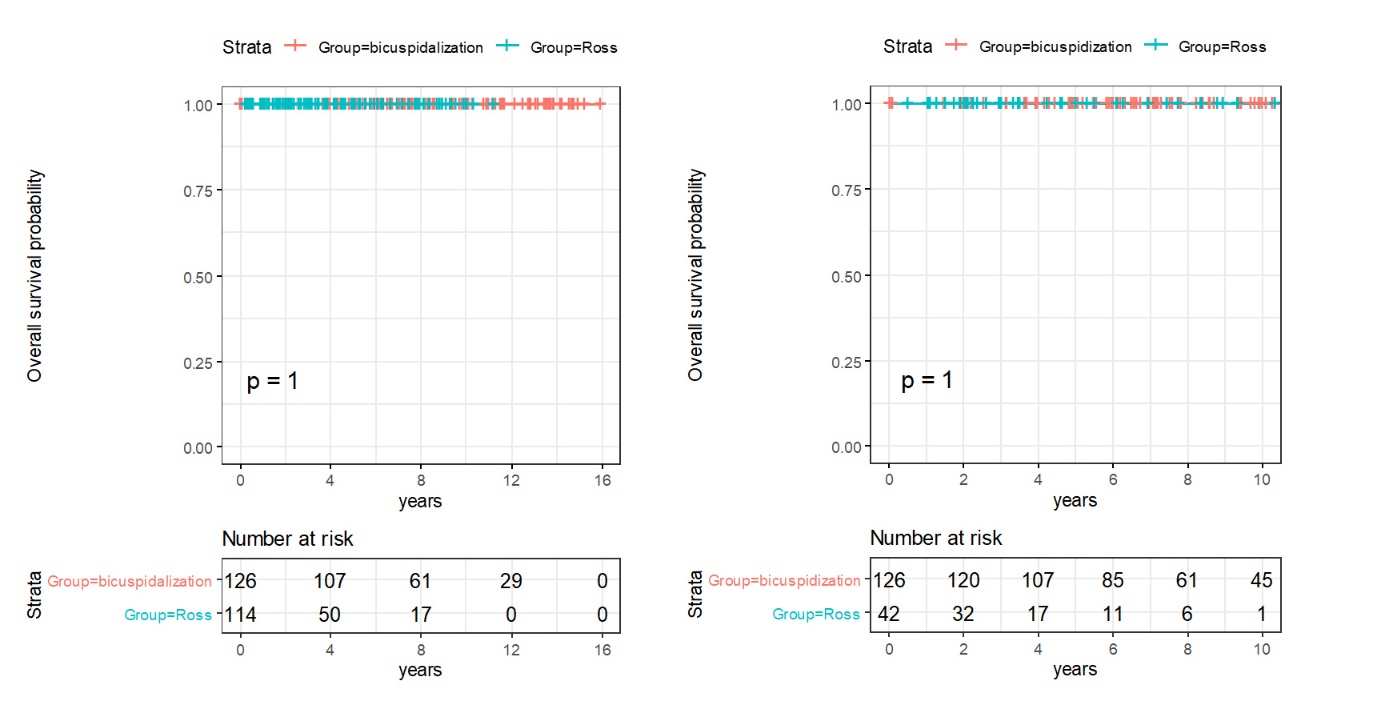

Supplement: Supplementary file 1 [file Data_Sheet_1.docx]
